# Genetic Diversity and Population Structure Analyses in Bitter Gourd (*Momordica charantia* L.) Based on Agro-Morphological and Microsatellite Markers

**DOI:** 10.3390/plants12193512

**Published:** 2023-10-09

**Authors:** K. N. Mallikarjuna, Bhoopal Singh Tomar, Manisha Mangal, Naveen Singh, Deepak Singh, Sachin Kumar, Avinash Tomer, Balraj Singh, Gograj Singh Jat

**Affiliations:** 1 Division of Vegetable Science, ICAR-Indian Agricultural Research Institute, New Delhi 110 012, India; mallikarjunkn1999@gmail.com (K.N.M.); bst_spu_iari@rediffmail.com (B.S.T.); manishamangal@rediffmail.com (M.M.); sachinchoudhary2096@gmail.com (S.K.); avin1140@gmail.com (A.T.); 2 Division of Genetics, ICAR-Indian Agricultural Research Institute, New Delhi 110 012, India; ns1genet@gmail.com; 3 ICAR-Indian Agricultural Statistical Research Institute, New Delhi 110 012, India; deepaksingh2112@gmail.com; 4 Sri Karan Narendra Agriculture University, Jobner 303 328, Rajasthan, India; drbsingh2000@gmail.com

**Keywords:** bitter gourd, diversity, population structure, microsatellite markers, gene flow

## Abstract

Bitter gourd (*Momordica charantia* L.) is an important vine crop of the Cucurbitaceae family and is well known for its high nutritional and medicinal values. However, the genetic variation remains largely unknown. Herein, 96 diverse bitter gourd genotypes were undertaken for diversity analysis using 10 quantitative traits, and 82 simple sequence repeat (SSR) markers. Out of 82 SSRs, 33 were polymorphic and the mean polymorphism information content (PIC) value was 0.38. Marker, JY-003 revealed a maximum (0.81) PIC value and, the number of alleles per locus ranged from 2 to 7 (average 3.46). The value of gene diversity showed the presence of a significant level of polymorphism among these genotypes. The unweighted pair group method (UPGMA) cluster analysis grouped the genotypes into two major clusters of which Cluster I comprised mostly small and medium-fruited genotypes of both *M. charantia* var. *charantia* and *M. charantia* var. *muricata*, whereas Cluster II included mostly long and extra-long fruited genotypes. Furthermore, these genotypes were divided into six distinct groups based on population structure analysis. The diversity analysis based on 10 quantitative traits revealed that earliness and high-yielding ability were exhibited by the predominantly gynoecious line DBGS-21-06 followed by DBGS-48-00. The principal component analysis (PCA) revealed that the first two components exhibited more than 50% of the total genetic variation. The present study deciphered a higher magnitude of agro-morphological and genetic diversity in 96 bitter gourd genotypes. Therefore, trait-specific genotypes identified in this study could be utilized in breeding programmes directed towards the development of improved cultivars and hybrids of bitter gourd.

## 1. Introduction

The bitter gourd (*Momordica charantia* L. 2n = 2x = 22) belongs to the family Cucurbitaceae. India is considered as the primary centre of origin of bitter gourd [1]. It was domesticated in eastern Asia, most likely in eastern India or southern China [2,3]. Tender fruits are used as vegetables and several processed products [4]. Predominantly, bitter gourd is monoecious (presence of both pistillate and staminate flowers on the same plant) however, gynoecious (presence of only pistillate flowers) sex form has also been reported from Japan, India, and China [5,6,7,8]. The botanical group, *M. charantia* var. *charantia* produces large fruits, and *M. charantia* var. *muricata*, produces small and round fruits those are used in curries, stuffed, fried, or pickled [1,9]. Fruits are an excellent source of nutrients such as carbohydrates, proteins, minerals, and vitamins, particularly ascorbic acid and iron, and are the most nutritious vegetable among cucurbits [9]. Apart from these nutrients, bitter gourd contains phenols, terpenes, saponins, and glucosinolates which contribute to its bitter taste [10]. The fruits are believed to be beneficial for the treatment of blood disorders, rheumatism, diabetes, and asthma [9].

Despite high nutritional and medicinal importance, the productivity of bitter gourd crops is low across the globe. Globally, the area under bitter gourd cultivation is increasing every year due to its high demand for consumption in various forms, such as cooked vegetables, juice, pickled, and processed products. The growth of traditional open-pollinated monoecious varieties and susceptibility of these commercial bitter gourd cultivars to virus complexes such as bitter gourd mosaic virus (BMV), tomato leaf curl virus (ToLCV), cucurbit aphid borne yellows virus (CABYV), and other devastating diseases such as downy mildew and powdery mildew and fruit fly as well are the major factors responsible for low productivity [11,12]. Thus, the evaluation, characterization, and utilization of a diverse set of bitter gourd germplasm is essential to enhance the genetic potential of cultivars.

The *Momordica* genus contains 59 species of which Africa has 47 different species, whereas Asia and Australia have only 12 different species [13]. Monoecious species are *M. charantia*, *M. charantia* var. *muricata,* and *M. balsamina*, whereas dioecious species are *M. dioica*, *M. sahyadrica*, *M. cochhinchinensis*, and *M. subangulata* [3,14,15,16]. Apart from *M. charantia* var. *charantia*, the wild species *M. charantia* var. *muricata* is widely cultivated in some parts of Asia [3]. Several morphological characters in these two species provide a relatively wide range of phenotypic variation, such as sex expression [7], growth habit, fruit shape, size, color, texture, and maturation [17,18,19]. In a nutshell, the presence of diverse species and traits within the genus provides more opportunities to the bitter gourd breeders for genetic improvement.

Genetic diversity has been an important factor and an essential pre-requisite for hybridization programmes to obtain progenies with desirable traits such as disease resistance, earliness, and quality traits [19,20]. Several researchers have evaluated the diversity in bitter gourd only based on phenotypic traits which has its limitations because most of the morphological characters are highly influenced by environmental factors and plant developmental stages [7,21,22,23]. The DNA-based markers are unaffected by environmental factors and shows high polymorphism [24]. The earlier conducted experiments in bitter gourd were based on the dominant marker system i.e., Randomly Amplified Polymorphic DNA (RAPD) [7,25], Inter Simples Sequence Repeats (ISSR) [22,25], Amplified Fragment Length Polymorphism (AFLP) [26] and involved only a few genotypes which do not provide sufficient information in the present context. SSR markers are ideal genetic markers that have gained significant importance in plant genetics and breeding due to desirable characteristics such as multi-allelic nature, co-dominant inheritance, abundance, high reproducibility, and high polymorphic compared to other markers [27,28,29], However, information on genetic diversity based on microsatellite markers is very limited in bitter gourd [19,30,31]. India is the primary centre of origin of bitter gourd and shows high genetic variation for fruit and growth types [19,23]. These important resources have hardly been exploited at the molecular level so far [31]. Therefore, the present study was carried out on genetic diversity and population structure analysis based on agro-morphological and SSR markers among 96 bitter gourd genotypes including wild species, *M. charantia* var. *muricata* collected from different regions of India and exotic collections.

## 2. Results

### 2.1. Per Se Performance of the Genotypes Based on Quantitative Traits

**Figure 1:**
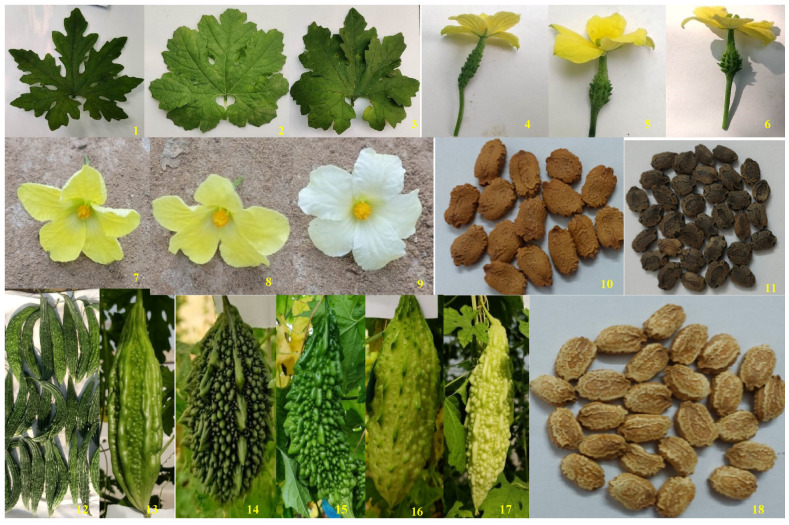
Visual observations of representative of 96 bitter gourd genotypes on the basis of various morphological traits; (**A**) Leaf shape: **1.** Multified **2.** Cordate **3.** Reniform; (**B**) Ovary length: **4.** Long, **5.** Medium, **6.** Small; (**C**) Flower colour: **7.** Dark yellow, **8.** Light yellow, **9.** White (DBGS-54-18); (**D**) Seed colour: **10.** Dark brown, **11.** Black, **18.** Whitish-brown; (**E**) Fruit length: **12.** Long, **13.** Medium, **14.** Small; (**F**) Fruit colour: **15.** Dark green, **16.** Light green, **17.** White.

Per se performance of ten quantitative traits among 96 bitter gourd genotypes are presented in Table 1 and visually observed diversity for plant, flower, and fruit characters are shown in Figure 1. Significant variation was observed for the traits studied in 96 bitter gourd genotypes. In the present study, white flowers in genotype DBGS-54-18 were recorded which is a unique character in bitter gourd (Figure 1, 9). DBGS-54-18 was developed using single plant selection from a segregating material. Fruits of DBGS-54-18 are medium, 12–14 cm long, 5.0–5.5 cm diameter with discontinuous narrow ridges.

The mean value for earliness traits i.e., the node number of the first female flower (NNFFF) was found to be lowest in Pusa Rasdar (9.14) followed by genotype DBGS-21-06 (9.27). The earliest flowering i.e., days to first female flower opening (DFFFO) was observed in predominantly gynoecious line DBGS-48-00 (38.62 days) which was at par with another predominantly gynoeciousline DBGS-21-06 (39.58) and Pusa Rasdar (40.20). DBG-7 (60.30 days) was the latest flowering genotype among the 96 genotypes evaluated. The lowest mean value for days to first male flower opening (DFMFO) was observed in Pusa Rasdar (36.14 days) followed by IC-444212 (36.47) and G-48 (37.11). Genotype G-35 (53.28 days) took the maximum number of DFMFO followed by G-22 (52.69 days). Significant variation was recorded for days to first fruit harvest (DFFH) which was found to vary from 45.68 days in genotype DBGS-48-00 to 73.40 days in DBG-7. Maximum fruit length (FL) at the edible maturity stage was recorded in Sel-2 (DBGS-2) (21.45 cm), whereas minimum FL was recorded in the genotype of *M. charantia* var. *muricata*, DBG-100 (4.65 cm). Maximum fruit diameter (FD) was observed in Pusa Rasdar (6.47 cm) whereas fruits of IC469518 recorded a minimum FD of 2.76 cm. The genotype IC-505638 had a maximum average fruit weight (AFW) of 88.63 g, and CBM-12 had a minimum AFW of 23.14 g. A maximum number of fruits per plant (NFPP) were recorded in IC68275 (44.40), followed by CBM-12 (42.36), IC858650 (42.15), and DBG-100-0 (41.36). Maximum yield per plant (YPP) was observed in predominantly gynoecious line DBGS-21-06 (2354.51 g) followed by DBGS-48-00 (2210.03 g). Genotype Sel-2 (DBGS-2) (193.20 cm) recorded maximum vine length (VL). The range for NNFFF, DFFFO, DFMFO, DFFH, FL, FD, AFW, NFPP, YPP and VL was 9.14–27.48, 38.62–60.30, 36.14–53.28, 45.68–73.40, 4.65–21.45, 2.76–6.23, 23.14–88.63, 12.36–44.40, 546.87–2354.51 and 118.63–171.28, respectively, which showed that these genotypes are highly diverse for the traits studied.

### 2.2. Cluster Analysis Based on Quantitative Traits

**Figure 2:**
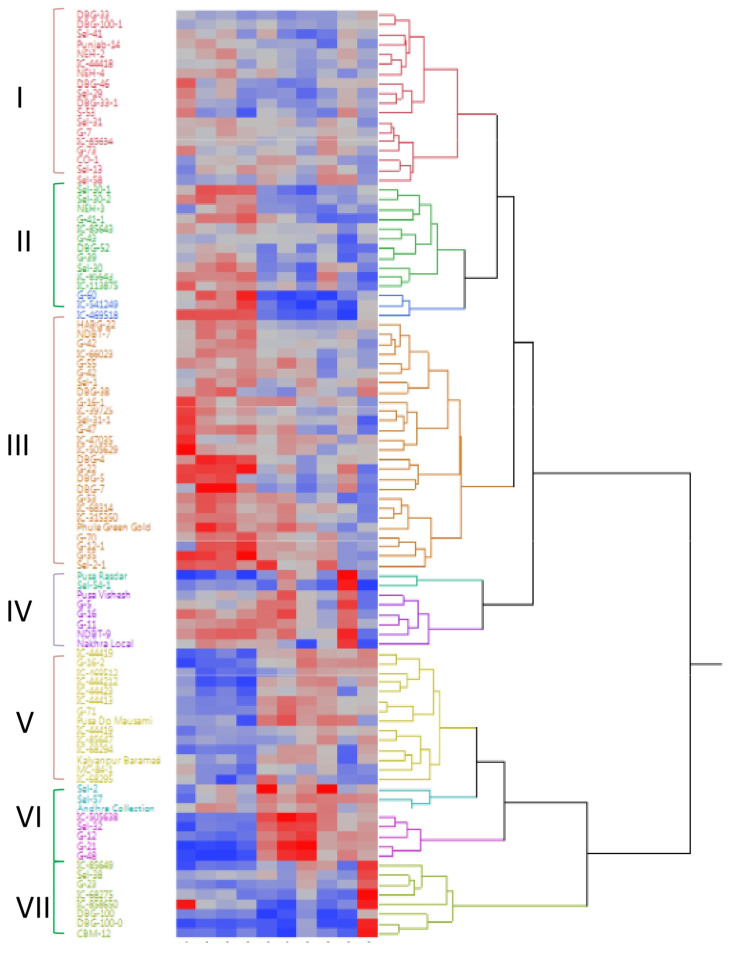
Clustering of dendrogram of 96 bitter gourd genotypes based on 10 quantitative traits.

The entire linkage cluster analysis dendrogram constructed for ten quantitative traits revealed diversity among the 96 bitter gourd genotypes and grouped all the genotypes into seven clusters (Figure 2). Out of 96 bitter gourd genotypes, 18 genotypes were placed in Cluster I which was further subdivided into sub-cluster-IA and IB. Sub-cluster IA had 11 genotypes whereas sub-cluster IB had 7 genotypes. Cluster II consisted of 14 genotypes and was further subdivided into IIA, IIB, and IIC sub-clusters. Sub-cluster IIA had 8 genotypes whereas sub-cluster IIB and IIC both had 3 genotypes in each sub-cluster. Cluster III consisted of 26 genotypes which was the largest group among all seven major clusters. Cluster III was further subdivided into IIIA, IIIB, and IIIC sub-clusters. Sub-cluster IIIA had 14 genotypes, IIIB comprised 4 genotypes whereas IIIC had 8 genotypes. Cluster IV contained 8 genotypes and was further subdivided into two sub-clusters; the first sub-cluster (IVA) comprised of 2 genotypes whereas the second sub-cluster (IVB) contained 6 genotypes. There were 17 genotypes in cluster V which were further divided into two sub-clusters first of which VA contained 14 genotypes and the second sub-cluster (VB) had 3 genotypes. Cluster VI had only 5 genotypes whereas Cluster VII comprised of 8 bitter gourd genotypes.

### 2.3. Correlation and Principal Component Analysis (PCA) for Phenotypic Traits

Pearson Correlation Matrix (Genotypic Correlations Matrix) among different quantitative traits in the bitter gourd is presented in Supplementary Table S3. Analysis of correlation revealed that YPP had a highly significant positive association with AFW (0.653), FL (0.591), VL (0.446), and NFPP (0.363) but had a highly significant negative correlation with DFFH (−0.569), and DFFFO (−0.560). The correlation between YPP and FD was positive (0.137), but it was not significant. Similarly, FL demonstrated a positive and statistically significant relationship with VL (0.611) and AFW (0.441). FD had a highly significant negative association with the NFPP (−0.408) and a positive correlation with AFW (0.520). The AFW displayed a highly significant positive relationship with the FL (0.754), FD (0.520), VL (0.441), and a negative association with the NFPP (−348). DFFFO had a highly significant negative correlation with the NFPP (−0.490), but it had a significant positive correlation with DFMFO (0.930), DFFH (0.801), and NNFFF (0.706). DFFH had a negative correlation (−0.475) with the NFPP, but a significant positive correlation with DFMFO (0.816) and NNFFF (0.614). DFFFO (−0.490), DFFH (−0.475), DFMFO (−0.413), FD (−408), and AFW (−0.348) all had a highly significant negative relationship. The correlation of the NFPP with VL was non-significant. The use of a simple correlation coefficient to assess character association may not provide an accurate picture of the relationship between yield and its components.

Path coefficient analysis was used to divide the genotypic correlations into direct and indirect effects to determine the relative relevance of ten characteristics. Estimates of the direct and indirect effects of different quantitative traits on YPP in the bitter gourd are presented in Supplementary Table S4. AFW had a maximum positive correlation (0.653) with YPP followed by FL (0.591) whereas DFFH (0.569) showed a highly significant but negative correlation. The direct effect of AFW (0.660) was also positive on YPP. Furthermore, it also contributed indirectly to a positive direction through FL (0.141), DFFFO (0.026), and VL (0.025). The negative indirect effect of AFW was also observed through NFPP (−0.167) and FD (−0.040). NFPP also had a positive direct effect (0.480) on YPP. It also contributed indirectly to a positive direction through DFFFO (0.104), FD (0.032), and DFFH (0.063) towards YPP. A negative indirect effect was recorded for AFW (−0.230) and FL (−0.015) on fruit YPP.

**Figure 3:**
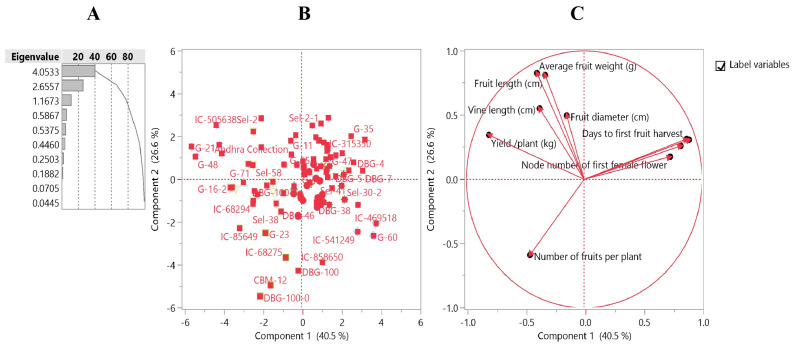
Principal Component Analysis (PCA) of 96 bitter gourd genotypes. (**A**) Summary plot based on Eigen-values depicting the percentage of variation explained by each principal component. (**B**) PCA biplot of different variables along with traits contributed. (**C**) PCA biplot of different variables used for the analysis of 96 bitter gourd genotypes.

The PCA analysis based on the Correlation matrix showed that the Eigenvalue of the first three PCs was >1 which explained 78.76% of the total variation (Supplementary Table S5). Further, the first two components (PC1 and PC2), based on summary plot analysis revealed >50% of the total variation i.e., 40.53% and 26.56%, respectively (Figure 3A). Additionally, variables such as DFFFO, DFFH, DFMFO, and NNFFF had a positive correlation with PC1 with a total variation of 40.53% and Eigenvalue 4.05 and these characters were found associated very closely with each other. DFFFO revealed the highest positive and NNFFF the lowest positive correlation with PC1. However, negatively correlated variables such as FL, FD, AFW, NF/P, YPP, and VL clustered together in the opposite direction. Two variables viz., AFW and FL, which had a positive correlation with PC2 but had a negative association with PC1 were plotted distantly from other variables (Figure 3C and Supplementary Table S5). VL and NFPP were positively correlated, whereas FD and AFW were negatively correlated with PC3. The major traits contributing to genetic diversity in PC4 were YPP and NFPP. Concurrently, among PC1, PC2, and PC3, analysis of the correlation of individual traits with principal components revealed that AFW had the highest positive correlation with PC2.

### 2.4. Diversity Statistics and Cluster Analysis Based on Microsatellite Markers

**Figure 4:**
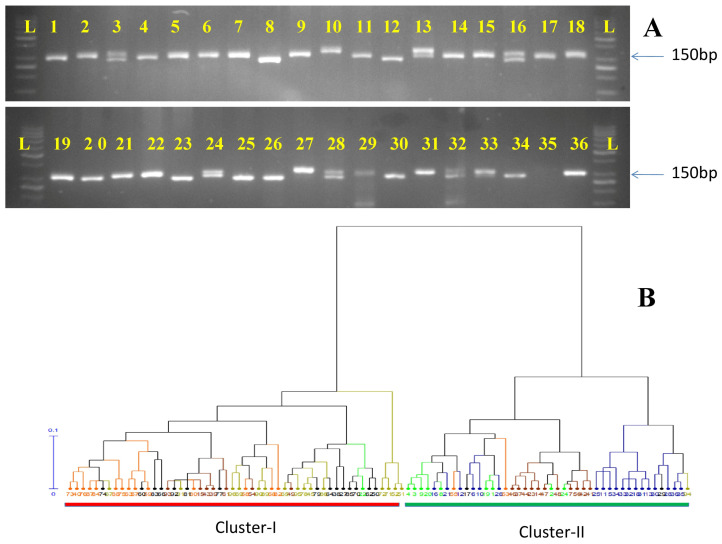
(**A**) Amplified profile of representative polymorphic marker S-24 (sample 1–36 listed in Table 2; L: 50 bp ladder); (**B**) UPGMA clustering based dendrogram of 96 bitter gourd genotypes (listed in Table 1) using 33 polymorphic SSR markers (Cluster 1 indicated with red colour, and Cluster II indicates with green colour).

Out of 82 SSRs screened in 96 bitter gourd genotypes, only 33 (41%) were found polymorphic and highly informative (Figure 4A and Table 2). The range of the alleles for polymorphic SSR markers were 50 to 290 bp. Marker, JY-003 showed the maximum variation for allele size (50–140 bp). A total of 112 alleles were detected based on 33 polymorphic SSR markers. The mean value was 3.46 alleles per locus (Table 2). The maximum number of alleles per locus was 7.0 alleles for JY-003, while the lowest (i.e., two alleles) for CMBR-57, CMBR-30, CMBR-31, JY-005, JY-008, McSSR-18, McSSR-11, McSSR-22, McSSR-47, McSSR-54, and S-33. The range of major allele frequency varied from 0.22 (JY003) to 0.94 (McSSR-54) with a mean of 0.68 at each locus. The frequencies of alleles were low, particularly for the loci with the higher number of alleles. Maximum heterozygosity (0.71) was recorded in marker, CMBR-57 and lowest (0.00) in CMBR-30, CMBR-31, JY-005, and McSSR-54 with an average value of 0.12 (Table 2). The number of genotypes obtained per locus ranged from 2 for CMBR-30, CMBR-31, JY-005, JY-008, McSSR-18, McSSR-11, McSSR-22, McSSR-47, and McSSR-54 to 17 for JY-003. Moreover, the polymorphic information content (PIC) value was used to measure the level of genetic diversity, which varied from 0.06 (S-33) to 0.81 (JY-003) with a mean value of 0.38. The value of gene diversity varied from 0.12 (McSSR-54) to 0.83 (JY-003), with an average value of 0.43 showing the presence of a high level of polymorphism among the 96 genotypes of bitter gourd (Table 2). In this study eight markers namely AVRDC_BG-66, AVRDC_BG-1, JY-003, JY-004, S-9, S-12, S-24,’ and S-32 showed PIC values >0.5 and were found highly informative in establishing genetic relationship among bitter gourd genotypes. Further, Hierarchical clustering grouped 96 bitter gourd genotypes using 33 SSR markers into two major clusters and further sub-clusters (Figure 4B).

Cluster I consisted of 52 genotypes and was divided into seven sub-clusters (1–7) having the majority of the *M. charantia* var. *charantia* genotypes. Out of seven sub-clusters, the first sub-cluster consisted of 9 genotypes including one *M. charantia* var. *muricata* (CBM-12) genotype and one released variety (Pusa Do Mausami). Sub-cluster II consisted of five advance and selection lines. Sub-cluster III consisted of 10 genotypes (G-11, IC-113875, G-43, DBG-52, Sel-30-2, G-21, Sel-13, NDBT-9, G-55, and IC-44419) having mostly medium-long fruited genotypes and showed superiority for earliness traits. Sub-cluster VI comprised eight genotypes with dark green fruit colour except NEH-4 having creamy white coloured fruit. Sub-cluster V also consisted of 10 genotypes which had mostly green coloured fruits with prominent spines on fruit surfaces. Sub-cluster VI consisted of only five genotypes having medium-long and light-green coloured fruits. Sub-cluster VII consisted of only four advanced breeding lines with a higher female: male (2:1) sex ratio. Major cluster II comprised 44 genotypes including *M. charantia* var. *charantia*, *M. charantia* var. *muricata*, and released varieties. This cluster was further divided into six sub-clusters (1–6). In sub-cluster I, out of nine genotypes, four genotypes (Sel-2 (DBGS-2), IC-44419, Pusa Rasdar, and IC-505638) were clustered together and showed superiority for all earliness and yield traits due to high female: male flower (2:1) ratio. In addition, Sel-2 (DBGS-2) present in this subcluster is also resistant to virus complex including Tomato Leaf Curl New Delhi Virus (ToLCNDV), and have extra-long (21.45 cm), dark green fruits with continuous sharp ridges and large long vine (Figure 1, 12). The other five genotypes produced light green to creamy white-coloured fruits except for DBG-38 which possesses dark green coloured fruits. Sub-cluster II consisted of seven genotypes mostly of indigenous collections. Sub-cluster III consisted of eight dark green coloured fruited genotypes having prominent tubercles except for DBG-5 which is having creamy white coloured fruits. Sub-cluster IV consisted of five genotypes of green and light-green colored fruits. Sub-cluster V comprised nine genotypes including one released variety Pusa Vishesh with light-green to glossy green coloured fruits and discontinuous ridges. In sub-cluster VI, a total of six genotypes represented extra small and medium size fruited genotypes.

### 2.5. Population Structure Analysis

**Figure 5:**
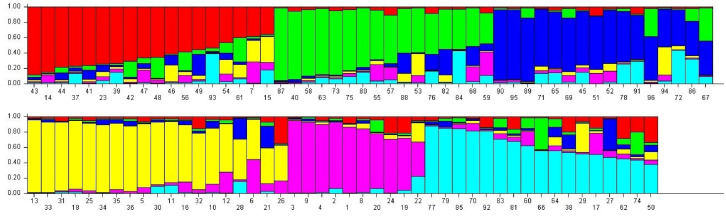
Population structure analysis using STRUCTURE v.2.3.4 and STRUCTURE HARVESTER v.o.6.94 based upon 33 SSR loci. Population structure bar plot of 96 bitter gourd genotypes. The red plot denotes Population I, the green plot denotes Population II, the blue plot denotes Population III, the yellow plot denotes Population IV, the purple plot denotes Population V, and the light blue plot denotes Population VI. Serial number (1–96) denotes the genotype names (listed in Table 1). Vertical value denotes the membership fraction.

STRUCTURE HARVESTER v.0.6.94 revealed six populations as ΔKvalue reached a peak at K = 6 (Figure 5). Population I included 18 genotypes of which most of the genotypes have medium-long fruit sizes and are late harvesting types. Population II & III comprised 16 genotypes each. Population II has mostly long-fruited genotypes but few genotypes recorded medium to long fruits with prominent tubercles, green, light green, and creamy colored fruits. Population III comprised mostly medium-long fruited genotypes with earliness character. Population IV included 19 genotypes comprising small, small to medium, and extra small-fruited types with prominent spines on the fruit surfaces. Population V included only 10 genotypes and was the smallest group comprising long to extra-long fruited genotypes. Population VI possessed 17 genotypes of mostly medium size fruits with vigorous vine and spiny fruit surfaces. Overall proportions of membership of the samples in each of the six populations were 0.19, 0.17, 0.17, 0.20, 0.10, and 0.18. The population structure analysis and the results specify that all the samples have an almost equal membership in their specific cluster. This result suggests that there has been a lot of gene flow in these genotypes.

### 2.6. Analysis of Molecular Variance (AMOVA) and Principal Component Analysis (PCA)

**Figure 6:**
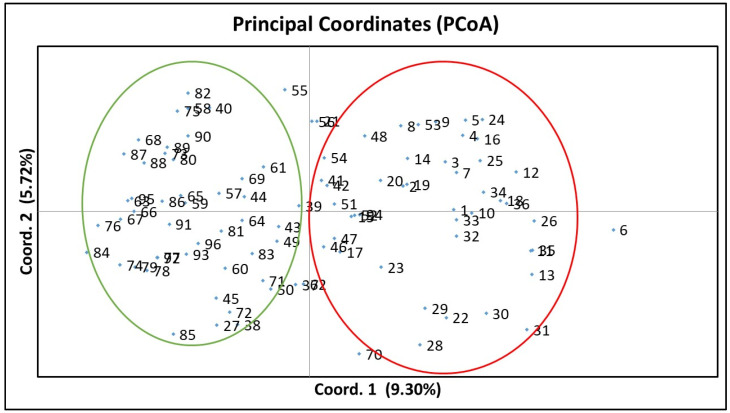
Principal Co-ordinate Analysis (PCoA) of 96 bitter gourd genotypes using Genealex v.6.5 [Group I indicate by the green colour and Group II indicates with red colour].

AMOVA and permutation test based on 999 permutations, states that there was 13% variation among the population whereas, individuals within the population showed 26% of the total variation (Table 3). Further, PCA also showed the genetic relationships among the bitter gourd genotypes studied. Percentages of variation of the top three PCs with their eigenvalues are presented in Table 4. Cumulatively, 20.24% of the variation was reported by the first three PCs with maximum variation in the first PC (9.30%) followed by the second PC (5.72%). The grouping of 96 bitter gourd genotypes into two major clusters based on UPGMA clustering was also supported by a PCA biplot (Figure 6). Group I (red circle) comprised all the genotypes of *M. charantia* except one genotype i.e., CBM-12; Group II (blue circle) comprised a mixture of *M. charantia* var. *charantia*, *M. charantia* var. *muricata*, released varieties, and advance breeding lines.

## 3. Discussion

Genetic diversity in bitter gourd based on morphological characters has been reported by several workers [21,25,30]. However, it has been observed that morphological characters are highly influenced by environmental factors [32,33,34]. Therefore, genetic diversity analysis based on SSR markers along with agro-morphological traits provides accurate information to detect closer relationships among the bitter gourd genotypes. Phenotypic variations for sex forms (gynoecious, predominantly gynoecious, and monoecious), and fruit characters (FL, diameter, weight, color, ridginess, etc.) could establish distinctness among the bitter gourd genotypes. Cluster analysis based on these traits revealed seven distinct clusters among the bitter gourd genotypes wherein these genotypes were differentiated based on flower characters, fruit size, shape, color, weight, length, diameter, sex forms, and earliness characters. DBGS-54-18 was a unique germplasm due to the presence of white flowers in the bitter gourd. This constitutes the first report of the occurrence of white flowers in bitter gourd which may be useful as a morphological marker in future breeding programmes. Variations for morphological characters among the clusters were also reported by [19,21,23,31]. However, some genotypes of *M. charantia* var. *muricata* were clustered together with cultivated *M. charantia* var. *charantia* which might be due to similarity in fruit types. Similarly, two predominantly gynoecious lines, DBGS-48-00 and DBGS-21-06 were clustered together with some monoecious genotypes which might be due to the similarity in earliness, sex ratio, and fruit traits of these two predominantly gynoecious lines with other monoecious genotypes. The grouping of gynoecious lines within the same cluster along with other monoecious genotypes was also reported by [19] and clear-cut differentiation within the wild species *M. charantia var. muricata* and other wild species were also reported by [16]. PCA biplot result showed that 78.76% of the total variation was reported by the first three PCs and this variation was higher than [19]. DFFFO revealed the highest positive correlation with PC1 whereas AFW had the highest positive correlation with PC2. Furthermore, analysis of Pearson’s correlation coefficient revealed that fruit YPP had a highly significant positive association with AFW, FL, VL, and NFPP, which was in accordance with [35,36].

Morphological markers alone are not sufficient to represent genetic diversity, but due to co-dominant inheritance, highly polymorphic, and high reproducibility nature of SSR markers, these were used in our study [27]. Out of 82 SSR markers, only 33 (41%) markers showed polymorphism, which was similar to the results of [19] but much higher than the value (24%) recorded by [30]. However, the average number of alleles per locus in our study was 3.46 alleles, which was higher than 2.80 alleles per locus as reported by [31], 3.1 alleles per locus for the Luoyang population and 2.6 alleles per locus for Guanzhou population as reported by [30] and 3.30 alleles per locus as reported by [19]. On the basis of PIC value, these 96 genotypes were divided into highly informative, reasonably informative, and slightly informative with their PIC value, >0.5, <0.5, >0.25, and <0.25, respectively. The present study showed that 15 SSRs (46%) were considered reasonably informative, and 6 (18%) were highly informative (Table 2). However, the PIC value ranged from 0.06 to 0.81 (mean 0.38); slightly higher than the PIC value (3.69) reported by [31] and 0.17 reported by [37], and lower than the PIC value (4.29) reported by [19]. The presence of a slightly high level of PIC in 96 bitter gourd genotypes of the present study might be due to high cross-pollination behaviour and similar findings were reported in other cross-pollinated crops such as cucumber [38,39].

The clustering analysis based on UPGMA grouped the 96 bitter gourd genotypes into two major clusters (cluster I and II), of which Cluster I comprised mostly *M. charantia* var. *charantia*. However, Cluster II comprised both *M. charantia* var. *charantia* and *M. charantia* var. *muricata* along with advanced breeding lines and released varieties which might be due to variation within the species concerning their adaptation and similarity for various traits [19]. The present finding is in accordance with the [19] as these genotypes were highly distinct from each other for growth, yield, and other quantitative traits and thus, designated into separate clusters [26]. Although, the genotypes of *M. charantia* var. *muricata* diverged from the *M. charantia* var. *charantia* based on agro-morphological and molecular markers clustering, due to a large number of sub-clusters (I-VII) on the basis of SSRs showed that environmental factors influence the development of genotypic variation. Further, the formation of major clusters and sub-clusters based on agro-morphological and SSR markers together was also reported by [31,37]. [21] grouped 38 bitter gourd genotypes into two major clusters using RAPD markers. [23,25] also grouped 38 bitter gourd genotypes into two major clusters using RAPD, ISSR, and AFLP markers. [31] grouped 26 bitter gourd genotypes into three major clusters using SSR markers. Recently, [19] grouped 51 bitter gourd genotypes into three clusters using SSR markers. No clear-cut correlation was established between phenotypic traits and SSR marker analysis. The reason behind this might be environmental effects on phenotypic traits and developmental stages of the plant. Furthermore, the SSR markers used in our study were random SSR markers, not EST-based markers.

Genetic diversity analysis, population structure patterns, genetic differentiation, and the level of admixture that exist between the populations or among the species are important for germplasm management and sustainable development [40]. In the present study, population structure analysis revealed the existence of six population groups (mixture of both *M. charantia* var. *muricata* and *M. charantia* var. *charantia* genotypes), exhibited more gene flow (admixture) from *M. charantia* var. *charantia* to *M. charantia* var. *muricata,* which enabled large scale cultivation *of M. charantia* var. *muricata* in several regions of India particularly eastern part of the country [19]. From population structure, it is well proved that *M. charantia* var. *muricata* was intermediate between cultivated and wild species, but it had a close relationship with the cultivated *M. charantia* var. *charantia* genotypes. Moreover, it might be one of the reasons that *M. charantia* var. *muricata* might be one of the parents in the evolution of *M. charantia* var. *charantia* [16]. Further, the divergence value of allele frequency >0.05 showed that the six groups of 96 genotypes were highly diverse which was also reported by [19]. AMOVA showed a significant variation in 96 bitter gourd genotypes, which was similar to the findings of [19].

PCA is used to find the best summary of the data using a limited number of PCs. PCA biplot formed two groups similar to the UPGMA cluster using SSR markers where bitter gourd genotypes were clearly distinguished from each other based on earliness and fruit characters. Such type of grouping pattern contradicts the grouping executed in the analysis of population structure which might be possible due to the implementation of the different algorithms in the STRUCTURE programme. Therefore, the grouping of 96 bitter gourd genotypes through UPGMA clustering, model-based analysis, and PCA was variable, showing the genetic variation in 96 genotypes at the DNA level [41,42].

Bitter gourd exhibits a high degree of cross-pollination [3] which may lead to admixtures in the population. High gene flow in the population opens the way for the development of new recombination events within the chromosomal level which favours further evaluation and development of new genetic variation [43]. From the present study, the genotypes such as DBGS-21-06, DBGS-48-00, Pusa Rasdar, and Sel-2 (DBGS-2) showed superior performance for most of the earliness and yield traits. Hence, these genotypes could be conserved in gene banks for their utilization in the development of early and high-yielding cultivars in bitter gourd. The polymorphic SSR markers identified in the present study will help in the genetic mapping and characterization of a large number of bitter gourd genotypes in the future.

## 4. Materials and Methods

### 4.1. Plant Material and Experimental Design

In this study, 96 morphologically and geographically diverse *M. charantia* var. *charantia* and *M. charantia var. muricata* genotypes were used including commercially released varieties and genotypes of exotic origin (Supplementary Table S1). These genotypes were maintained through continuous self-pollination at the research farm of the Division of Vegetable Science, ICAR-Indian Agricultural Research Institute, New Delhi. The field experiment was conducted in a Randomized Block Design (RBD) with three replications.

### 4.2. Field Experimental Conditions

Seeds were sown on both sides of the channel, with 3 m spacing between channels and 60 cm between plants, with 90 cm wide irrigation channels. The soil of the experiment was sandy loam inceptisol. The crop was sown on the third week of February 2022. To raise a healthy crop, the recommended dose of fertilizers, cultural practices, and proper plant protection measures were followed. The doses of fertilizers were 80 kg Nitrogen, 60 kg Phosphorus, and 60 kg Potash per hectare. The full dose of Phosphorus and Potash and half dose of Nitrogen were applied at the time of the last ploughing. The remaining half dose of Nitrogen was applied into two split doses through top-dressing at 30 and 45 days after sowing of the seeds. The plants on the border of the channels were excluded and discarded and five competitive and random plants were taken for recording phenotypic observations on 10 important quantitative traits. To record data on fruit traits, fruits were harvested at the marketable stage.

### 4.3. Morphological Characterization and Cluster Analysis

Observations were recorded on 96 bitter gourd genotypes for different agro-morphological traits at different developmental stages from flowering to fruit maturity. Ten quantitative traits viz., NNFFF, DFFFO, DFMFO, DFFH, FL, FD, AFW, NFP, YP, and VL were undertaken for observations (Table 1). A phylogenetic dendrogram was constructed based on the hierarchical clustering of D^2^ values [44].

### 4.4. PCA for Phenotypic Traits

The genotypic and phenotypic correlations were determined using an analysis of variance and covariance matrix where total variability was split into replications, genotypes, and errors [45]. Statistical significance was also determined at *p* < 0.05 and *p* < 0.01. The measured correlation coefficients were compared [46] and tabulated values at (n-2) degree of freedom, where “n” denotes the number of genotypes to confirm their significance. A PCA biplot was constructed for 10 quantitative traits studied in 96 genotypes using the SAS package [47] to reduce the dimensionality of a data set and the estimation of the contribution of each quantitative trait. PCA biplot revealed the schematic representation of both individual genotypes and different variables in a two-dimensional chart. Additionally, a summary plot was also constructed to represent the percentage of variation explained by individual components [48].

### 4.5. DNA Extraction and PCR Amplification

Leaf samples were collected from 30 days old plants of each genotype for DNA extraction using the modified CTAB method [49] with slight modifications. The quality and quantity of extracted DNA samples were determined using 2% agarose gel. The concentration of DNA was adjusted to 20–50 ng/µL and then stored at −20 °C for further usage. The PCR reaction mixture consisted of 1 µL diluted DNA sample, 0.5 µL of each forward and reverse primer, 3 µL nuclease-free water, and 5 µL master mix (one PCR^TM^ of Genedirex, Inc. Germany) in 10 µL reaction volume. For PCR amplification, Eppendorf Mastercycler was used following the PCR steps of; initial denaturation at 95 °C for 5 min followed by 35 cycles at three conditions (i) 95 °C for 30 s, (ii) at an annealing temperature for a particular primer (Supplementary Table S2) for 45 s, (iii) 70 °C for 60 s and a final extension at 72 °C for 10 min followed by 4 °C cooling at an infinite time. Amplified PCR products were separated on 3% agarose gel electrophoresis in 1X TAE buffer (pH 8.0). The gel was run at 120 mA voltage for 2.5 h and gel pictures were visualized and captured under a gel documentation system (Alpha imager HP, Proteinsimple^TM^, Santa Clara, CA, USA) using 50 bp DNA ladder (GeNie^TM^) to compare the fragment sizes. A total of 82 SSRs markers were screened, out of these, 33 polymorphic markers were used for genetic diversity and population structure analysis [19,31] (Supplementary Table S2).

### 4.6. Genetic Diversity and Population Structure Analysis

The amplified polymorphic bands of each primer were used for the scoring and analysis. The scored markers were represented by a binomial (0/1) matrix, which was the basic data structure. PIC was calculated using the software Power Marker v.3.5 [50] using the following formula as suggested by [51].

Genetic diversity parameters such as gene diversity, heterozygosity, number of alleles, and major allele frequency were calculated using the tool Power Marker v3.5 and pairwise genetic similarity between the genotypes using Jaccard’s similarity coefficient was developed using NTSYSpc v.2.02 [52]. AMOVA and PCoA were analyzed using GenAlEx v6.5 [53]. Population structure was analyzed using the Bayesian model-based clustering approach implemented in STRUCTURE v2.3.4 software [54]. The software programme burn-in period was set at 10,000 lengths followed by 100,000 Markov Chain Monte Carlo (MCMC) repetitions and a range of putative *k* value was kept from *k* = 1 to *k* = 10, each having 15 iterations/replications for running each *k* independently. The optimum value of *k* for the entire population was determined according to the simulation method of delta *K* (Δ*K*) value [55] using the web-based software programme STRUCTURE HARVESTER v.0.6.94 [56].

## 5. Conclusions

A considerable amount of variation was present among 96 bitter gourd genotypes as evident from morphological and microsatellite markers-based diversity analyses. Genotypes of the present study were quite distinct from each other as indicated by a broad range of similarity coefficients. Grouping genotypes into two major clusters and further into sub-clusters will be highly informative for the bitter gourd breeders for genetic enhancement and to setup its core collections. Thus, diversity analyses based on morphological and molecular markers have identified superior genotypes and highly polymorphic SSR markers which will be useful for mapping or tagging the economically important traits. The present study has also provided excellent information on the population structure and genetic diversity of 96 bitter gourd genotypes which will be highly useful in the bitter gourd improvement programme for the development of trait-specific cultivars for earliness, yield, and other economic traits.

## Figures and Tables

**Table 1:** Mean performance of 96 diverse bitter gourd genotypes for 10 quantitative characters.

S. No.	GENOTYPES	NNFFF	DFFFO	DFMFO	DFFH	FL (cm)	FD (cm)	AFW (g)	NFPP	YPP (g)	VL (cm)
1	DBG-33	17.30	47.22	40.66	57.55	10.22	3.98	40.25	25.14	978.25	135.22
2	Sel-31	18.57	51.33	43.78	63.44	12.47	4.10	57.22	18.32	1002.30	152.33
3	IC-44419	11.06	42.36	37.52	53.64	13.22	4.31	58.63	30.14	1623.47	149.54
4	Sel-2 (DBGS-2)	13.14	50.36	42.60	62.47	21.45	3.81	57.84	25.47	1521.30	193.20
5	HABG-22	18.64	54.63	47.30	63.87	11.47	3.87	47.32	24.57	987.52	137.22
6	NDBT-7	18.20	54.64	48.32	62.38	12.33	4.33	52.38	20.45	1123.35	139.47
7	DBG-4	20.34	58.97	49.65	70.41	12.36	4.28	52.47	23.47	934.25	142.87
8	Sel-30-1	18.55	56.35	48.63	67.51	9.35	3.64	35.40	22.55	675.55	135.66
9	Pusa Rasdar	9.14	40.20	36.14	52.65	9.45	6.47	67.84	18.69	1244.60	132.44
10	IC-469512	15.75	41.27	38.55	51.63	9.54	4.35	48.75	25.40	1489.63	152.47
11	IC-444212	15.02	40.32	36.47	49.33	13.77	3.84	56.36	26.84	1542.36	161.47
12	IC-44423	15.78	42.65	37.66	53.25	12.87	3.33	52.47	23.37	1365.00	154.33
13	G-53	21.06	56.36	45.29	66.80	14.98	3.22	61.33	17.65	988.32	142.55
14	Phule Green Gold	20.35	58.90	45.24	67.88	15.22	3.45	72.33	22.00	1467.32	138.65
15	IC-44419	12.63	46.32	41.20	56.32	10.97	4.14	51.38	24.33	1235.47	153.39
16	CO-1	14.55	50.32	43.22	61.05	14.35	3.67	61.20	18.54	1142.33	146.67
17	IC-85643	19.87	50.33	46.35	58.44	12.10	3.47	51.37	22.63	1128.61	146.58
18	DBG-100-1	15.80	48.65	42.35	59.63	12.35	4.12	44.62	23.34	1023.25	134.74
19	IC-85649	10.14	43.60	39.54	51.28	11.30	3.24	42.50	39.50	1544.38	154.36
20	IC-505638	11.68	44.28	39.65	52.38	18.32	4.21	88.63	25.32	2136.54	163.24
21	NEH-3	13.71	50.24	47.65	63.35	9.54	3.87	42.67	18.61	865.32	127.63
22	IC-68295	17.48	45.36	40.30	47.19	16.87	3.47	45.56	17.45	1425.56	136.54
23	IC-85647	14.77	47.63	40.87	55.80	9.65	3.79	46.30	28.40	1241.28	159.87
24	Sel-1	17.15	54.30	47.50	62.17	10.42	4.25	51.33	28.63	1497.63	142.56
25	Pusa Vishesh	15.45	49.36	42.58	61.50	14.30	5.20	75.30	16.30	1210.20	140.20
26	G-16-2	9.76	42.14	39.54	50.28	12.30	4.78	55.32	29.65	1698.66	156.34
27	DBGS-54-18	18.48	54.30	46.28	63.87	13.34	5.01	57.40	19.54	1147.26	132.84
28	DBG-100	13.56	45.32	40.22	52.66	4.65	3.02	28.54	24.69	932.15	118.63
29	DBG-46	23.29	47.60	41.20	61.84	9.45	4.21	36.44	24.50	863.00	146.52
30	Sel-30	18.60	53.47	45.60	66.84	8.53	4.31	42.10	17.50	702.30	152.30
31	DBG-100-0	9.94	41.36	38.22	48.36	6.33	2.89	25.30	41.36	1032.55	120.24
32	Sel-54-1	13.38	46.32	41.20	54.36	7.11	6.23	62.38	12.36	863.25	124.20
33	Sel-38	15.48	48.33	42.58	59.22	13.11	3.57	43.58	37.21	1387.22	138.42
34	G-16-1	24.88	52.80	46.32	60.28	15.20	4.23	66.35	18.63	1121.33	151.20
35	IC-85634	18.32	49.65	44.25	61.38	11.28	4.14	53.39	22.24	1078.68	158.61
36	Sel-32	9.88	44.65	38.95	52.62	16.84	4.21	78.69	27.68	1987.33	162.35
37	G-16	22.55	53.28	46.38	61.28	16.23	5.21	77.32	18.62	1236.30	138.44
38	IC-68275	16.82	47.36	39.47	58.63	8.65	3.21	35.68	44.40	1145.32	128.63
39	G-55	20.87	52.36	46.28	60.38	13.28	4.16	68.47	19.57	1340.20	130.25
40	G-41-1	18.33	54.32	49.18	65.38	13.98	3.21	54.32	16.34	947.33	124.87
41	G-12	13.44	43.25	39.25	50.14	15.48	4.87	74.25	28.36	2070.22	152.39
42	G-60	18.01	56.32	52.17	64.38	6.21	3.41	24.30	21.30	648.32	117.25
43	NDBT-9	20.36	54.87	48.65	67.32	14.21	6.11	70.78	16.24	1140.28	146.27
44	Sel-41	19.30	49.52	40.66	61.88	13.44	4.38	38.96	21.11	732.41	129.45
45	Sel-29	21.38	48.60	40.18	61.70	9.32	4.30	43.25	19.55	754.20	135.21
46	DBG-5	24.74	56.28	42.18	69.35	10.30	4.65	51.38	17.63	836.21	139.50
47	G-42	18.36	52.50	47.60	64.80	12.00	4.20	52.72	18.23	1250.56	143.25
48	G-70	18.87	54.68	49.60	65.33	14.20	3.79	56.34	19.43	1143.26	157.39
49	NEH-2	17.77	51.43	42.85	64.35	12.10	4.46	49.57	20.64	978.20	141.35
50	IC-44418	17.32	49.68	41.25	61.32	12.54	4.27	48.93	19.67	956.38	137.54
51	Sel-2-1	23.61	55.28	46.18	67.35	19.57	3.65	53.87	20.39	1241.28	171.28
52	G-7	18.81	49.63	44.35	63.22	12.54	4.21	52.11	21.11	1147.11	148.33
53	Sel-58	14.69	47.23	41.36	58.36	13.87	4.87	51.63	19.36	976.38	161.20
54	NL-39	16.61	51.30	46.88	63.22	13.98	4.02	56.54	23.54	1322.64	132.01
55	DBG-38	19.25	54.28	42.17	67.38	10.28	3.71	40.17	31.47	1347.50	142.10
56	G-12-1	14.44	56.32	51.34	67.98	14.25	3.62	62.32	20.31	1262.20	153.20
57	DBG-7	16.65	60.30	47.28	73.40	9.10	4.94	46.38	16.74	796.28	137.58
58	NEH-4	19.35	51.47	43.17	64.35	13.62	3.52	50.30	21.30	934.20	141.28
59	Sel-57	12.09	49.35	41.87	62.38	15.84	4.92	61.38	29.10	1698.30	162.47
60	IC-68294	12.04	44.36	39.33	52.30	11.36	3.14	62.31	26.30	1465.35	145.22
61	Sel-13	13.65	51.32	46.28	60.29	13.28	4.10	56.32	18.63	1036.38	157.68
62	G-23	14.50	44.50	40.32	51.32	9.54	3.89	42.30	34.25	1325.68	134.27
63	G-73	21.47	48.63	42.33	59.67	12.66	4.33	53.66	20.11	978.33	158.39
64	IC-39725	24.65	54.28	45.80	61.44	11.20	4.14	54.22	20.00	1024.30	135.28
65	G-22	24.46	57.65	52.69	69.54	11.14	5.14	61.32	16.35	932.30	143.67
66	IC-68314	19.82	56.28	44.20	65.30	14.00	4.12	66.41	17.32	1230.22	152.68
67	IC-85643	21.17	53.28	48.35	64.51	9.14	3.63	50.32	14.36	734.25	163.35
68	IC-315350	21.02	54.31	45.70	65.74	13.65	3.89	65.74	17.60	1134.50	142.66
69	G-35	25.57	57.68	53.28	67.35	14.20	3.64	57.39	18.33	1368.65	154.34
70	Sel-31-1	25.17	52.31	44.65	60.17	12.47	4.35	57.32	14.78	1104.36	142.14
71	IC-44413	14.55	44.36	38.74	53.68	14.57	4.10	70.21	23.30	1560.32	152.36
72	G-47	24.26	53.24	48.32	65.32	13.35	4.27	65.30	16.52	988.74	131.28
73	CBM-12	12.75	43.65	40.14	49.36	6.25	3.10	23.14	42.36	1020.36	132.04
74	Kalyanpur Baramasi	17.70	45.87	39.64	57.84	13.74	3.12	62.38	24.67	1428.97	146.37
75	Pusa Do Mausami	15.43	46.58	37.14	57.68	16.10	5.02	75.68	19.30	1438.30	165.24
76	G-71	14.61	44.60	40.28	53.28	14.10	4.13	71.25	24.36	1467.58	153.27
77	DBGS-21-06	9.27	39.58	38.21	46.32	16.42	4.68	85.33	27.32	2354.51	152.39
78	DBGS-48-00	9.89	38.62	37.11	45.68	15.32	4.96	81.36	25.63	2210.03	146.37
79	Punjab-14	18.73	52.36	43.36	60.58	10.35	4.21	47.49	18.64	1032.44	126.58
80	DBG-52	17.38	51.28	42.88	60.74	8.11	3.17	53.24	16.24	834.11	142.45
81	Sel-30-2	21.12	55.87	46.17	63.41	9.32	3.58	40.21	21.47	874.20	139.14
82	IC-541249	17.37	52.32	50.66	64.22	8.30	2.88	28.33	23.05	546.87	128.63
83	IC-469518	23.95	56.38	49.27	68.32	7.98	2.76	31.20	23.22	678.58	125.56
84	G-5	18.48	50.30	44.60	57.63	16.35	5.84	69.36	16.32	1236.65	132.65
85	MC-84-1	18.83	45.39	38.47	53.98	12.32	3.41	52.36	18.47	1020.47	147.36
86	IC-47035	25.63	49.33	45.62	58.64	12.14	3.68	63.34	24.67	1432.68	151.29
87	S-53	21.68	45.18	37.65	56.17	12.04	4.88	50.27	19.67	932.74	129.54
88	Andhra Collection	17.87	53.20	42.36	63.47	15.13	3.82	56.74	26.32	1463.28	163.87
89	DBG-33-1	20.19	47.88	39.87	56.74	11.30	4.32	42.30	20.74	896.44	138.69
90	Nakhra Local	17.63	53.47	44.63	62.87	13.47	5.86	47.38	13.87	603.98	142.38
91	G-11	19.47	52.62	48.20	64.38	16.24	5.26	64.35	21.28	1235.62	138.56
92	IC-113875	23.27	50.32	46.32	63.35	11.20	3.68	57.32	16.39	921.05	154.32
93	G-43	16.56	47.65	43.29	59.81	12.34	3.04	53.27	21.36	1141.36	137.61
94	IC-505629	27.48	52.38	44.17	61.38	12.57	4.11	62.87	23.35	1314.25	135.29
95	IC-858650	26.84	49.65	42.87	57.33	6.47	3.52	25.63	42.15	963.35	126.41
96	G-39	16.36	51.67	43.66	64.68	8.33	3.10	46.94	19.32	985.66	143.68
	**Mean**	**17.77**	**50.00**	**43.54**	**60.13**	**12.27**	**4.09**	**54.03**	**22.66**	**1177.39**	**143.95**
	**Minimum**	**9.14**	**38.62**	**36.14**	**45.68**	**4.65**	**2.76**	**23.14**	**12.36**	**546.87**	**118.63**
	**Maximum**	**27.48**	**60.30**	**53.28**	**73.40**	**21.45**	**6.23**	**88.63**	**44.40**	**2354.51**	**171.28**
	**SE (m)**	**0.663**	**1.806**	**0.880**	**1.049**	**0.747**	**0.299**	**1.275**	**1.145**	**18.719**	**2.083**
	**SE(d)**	**0.938**	**2.553**	**1.244**	**1.483**	**1.056**	**0.423**	**1.804**	**1.619**	**26.473**	**2.949**
	**CD0.05**	**1.84**	**5.00**	**2.44**	**2.91**	**2.07**	**0.83**	**3.54**	**3.17**	**51.89**	**5.78**
	**CD0.01**	**2.42**	**6.58**	**3.20**	**3.82**	**2.72**	**1.09**	**4.65**	**4.17**	**68.19**	**7.59**
	**CV%**	**6.46**	**6.26**	**3.50**	**3.02**	**10.55**	**12.68**	**4.09**	**8.75**	**2.75**	**2.51**

NNFFF: Node number of first female flower; DFFFO: Days to first female flower opening; DFMFO: Days to first male flower opening; DFFH: Days to first fruit harvest; FL: Fruit length (cm); FD: Fruit diameter (cm); AFW: Average fruit weight (g); NFPP: Number of fruits per plant (No.); YPP: Yield per plant(g); VL: vine length(cm).

**Table 2:** Genetic diversity, heterozygosity, polymorphic information content (PIC), major allele frequency, and number of alleles from analysis of polymorphic SSR markers obtained through Power Marker analysis.

Marker	Major Allele Frequency	Allele No.	Gene Diversity	Heterozygosity	PIC
**AVRDC_BG-83**	0.79	5	0.35	0.14	0.33
**AVRDC_BG-66**	0.55	5	0.62	0.18	0.57
**AVRDC_BG-74**	0.78	4	0.37	0.12	0.34
**AVRDC_BG-1**	0.40	4	0.66	0.14	0.60
**AVRDC_BG-2**	0.87	4	0.24	0.04	0.23
**AVRDC_BG-95**	0.53	4	0.52	0.01	0.41
**BGSSR-08**	0.48	3	0.56	0.03	0.47
**CMBR-57**	0.64	2	0.46	0.71	0.36
**CMBR-22**	0.69	4	0.48	0.17	0.43
**CMBR-30**	0.92	2	0.15	0.00	0.14
**CMBR-31**	0.84	2	0.26	0.00	0.23
**JY003**	0.22	7	0.83	0.36	0.81
**JY-004**	0.48	4	0.66	0.05	0.61
**JY-005**	0.73	2	0.39	0.00	0.31
**JY-008**	0.83	2	0.28	0.01	0.24
**JY-009**	0.62	3	0.54	0.06	0.48
**JY-011**	0.68	3	0.46	0.06	0.39
**McSSR-18**	0.90	2	0.18	0.03	0.16
**McSSR-11**	0.87	2	0.23	0.07	0.20
**McSSR-22**	0.92	2	0.14	0.09	0.13
**McSSR-47**	0.71	2	0.41	0.49	0.33
**McSSR-54**	0.94	2	0.12	0.00	0.11
**N-1**	0.72	3	0.43	0.10	0.38
**N-6**	0.84	5	0.28	0.08	0.26
**N-9**	0.79	5	0.35	0.02	0.33
**S-9**	0.57	4	0.57	0.15	0.51
**S-12**	0.51	4	0.64	0.25	0.58
**S-13**	0.79	4	0.35	0.14	0.33
**S-20**	0.49	3	0.556	0.02	0.46
**S-24**	0.35	6	0.72	0.13	0.66
**S-32**	0.43	4	0.69	0.27	0.64
**S-33**	0.97	2	0.06	0.02	0.06
**S-26**	0.76	4	0.40	0.06	0.34
**Mean**	**0.68**	**3.45**	**0.43**	**0.12**	**0.38**

**Table 3:** Summary of Analysis of molecular variance (AMOVA).

Source	df	SS	MS	Est. Var.	Var.%	Statistic	Value	Probability
Among Population	5	214.77	42.95	1.01	13	Fst	0.13	0.001
Among Individuals	90	990.85	11.01	4.52	60	Fis	0.70	0.001
Within Individuals	96	189.00	1.97	1.97	26	Fit	0.74	0.001
Total	191	1394.62	-	7.49	100	-	-	-

**Table 4:** Percentage of variation explained by principal components through analysis of PCA biplot on the basis of molecular markers.

Axis	1	2	3
Variation %	9.30	5.72	5.22
Cumulative variation %	9.30	15.02	20.24

## Data Availability

Not applicable.

## Supplementary Materials

The following supporting information can be downloaded at: https://www.mdpi.com/article/10.3390/plants12193512/s1, Table S1: List of 96 bitter gourd (*Momordica charantia* L.) genotypes, sources and salient features; Table S2: Forward and reverse sequence of 33 SSR markers used in the study; Table S3: Pearson Correlation Matrix (Genotypic Correlations Matrix) among different quantitative traits in 96 bitter gourd genotypes; Table S4: Estimates of direct and indirect effects of different quantitative traits on fruit YPP in bitter gourd genotypes; Table S5: Corresponding Eigen vectors for different principal components (PCs) based on analysis of phenotypic traits.
