# Supplementary material for: Genetic Diversity and Population Structure Analyses in Bitter Gourd (Momordica charantia L.) Based on Agro-Morphological and Microsatellite Markers
Source: Plants (Basel). 2023 Oct 9;12(19):3512. doi: 10.3390/plants12193512 (PMC10574847; doi:10.3390/plants12193512)
Supplement: Supplementary file 1 [file plants-12-03512-s001.zip › plants-2402911-supplementary.pdf]

Supplementary Material

Table S1. List of 96 bitter gourd (*Momordica charantia* L.) genotypes, sources and salient features.

No.	Accessions name	Salient features	Taxon/Species	Sources
1	DBG-33	Green, extra-long fruits with discontinuous ridges, medium vine	A	West Bengal, India
2	Sel-31	Dark green, medium long fruits with continuous ridges	A	Philippines
3	IC-44419	Light green, medium long fruits with discontinuous ridges and spiny surface	A	NBPGR, New Delhi
4	Sel-2	Dark green, extra-long fruits with continuous sharp ridges, large long vine	A	IARI, New Delhi
5	HABG-22	Creamy white, medium fruits with continuous ridges	A	IARI, New Delhi
6	NDBT-7	Dark green, medium long fruits with discontinuous ridges	A	Faizabad, India
7	DBG-4	White, long fruits with continuous ridges, medium vine	A	IARI, New Delhi
8	Sel-30-1	Light green, extra-long fruits with discontinuous ridges	A	Philippines
9	Pusa Rasdar	Dark green, capsicum shaped, medium with continuous smooth ridges, medium vine	A	IARI, New Delhi
10	IC-469512	Dark green, medium long fruits with discontinuous ridges and spiny surface	A	NBPGR, New Delhi
11	IC-444212	Green, medium long fruits with discontinuous ridges and spiny surface	A	NBPGR, New Delhi
12	IC-44423	Creamy white, long fruits with discontinuous ridges and prominent tubercles	A	NBPGR, New Delhi
13	G-53	Green, medium long fruits with discontinuous ridges	A	IARI, New Delhi
14	Phule Green Gold	Dark green, medium long fruits with discontinuous ridges and spiny surface	A	Maharashtra, India
15	IC-44419	Light green, medium long fruits with discontinuous ridges	A	NBPGR, New Delhi
16	CO-1	Green, medium long fruits with discontinuous ridges and spiny surface	A	TNAU, Coimbatore
17	IC-85643	Green, medium long fruits with discontinuous ridges and spiny surface	A	NBPGR, New Delhi
18	DBG-100-1	Green, small fruits with discontinuous ridges and spiny surface	B	IARI, New Delhi
19	IC-85649	Green, small fruits with discontinuous ridges and spiny surface	B	NBPGR, New Delhi
20	IC-505638	Green, long fruits with discontinuous ridges and prominent tubercles	A	NBPGR, New Delhi
21	NEH-3	Green, medium long fruits with discontinuous ridges	A	Manipur, India
22	IC-68295	Light green, long fruits with discontinuous ridges and prominent tubercles	A	NBPGR, New Delhi
23	IC-85647	Dark green, small fruits with continuous ridges and smooth surface	B	NBPGR, New Delhi
24	Sel-1	Light green, long fruits with continuous ridges, medium vine	A	IARI, New Delhi
25	Pusa Vishesh	Glossy green, long fruits with continuous ridges, medium vine	A	IARI, New Delhi
26	G-16-2	Dark green, medium long fruits with discontinuous ridges, spiny surface and prominent tubercles	A	IARI, New Delhi
27	DBGS-54-18	Light green, medium long fruits with discontinuous narrow ridges, white flowers	A	IARI, New Delhi
28	DBG-100	Dark green, extra small fruits with discontinuous ridges and spiny surface	B	IARI, New Delhi

29	DBG-46	Dark green, medium long fruits with discontinuous ridges, spiny surface and prominent tubercles	A	West Bengal, India
30	Sel-30	Glossy green, small fruits with continuous ridges and smooth surface	A	China
31	DBG-100-0	Dark green, extra small fruits with prominent tubercles and spiny surface	B	IARI, New Delhi
32	Sel-54-1	Dark Green, medium long fruits with continuous ridges and smooth surface	A	Taiwan
33	Sel-38	Green, medium long fruits with discontinuous ridges, medium vine	A	Lao
34	G-16-1	Green, long fruits with discontinuous ridges and spiny surface prominent tubercles	A	IARI, New Delhi
35	IC85634	Green, medium fruits with continuous ridges	A	NBPGR, New Delhi
36	S-32	Green, medium long fruits with continuous ridges	A	Philippines
37	G-16	Dark green, medium long fruits with discontinuous ridges, spiny surface and prominent tubercles	A	IARI, New Delhi
38	IC-68275	Green, small fruits with discontinuous ridges, spiny surface and prominent tubercles	B	NBPGR, New Delhi
39	G-55	Dark green, medium long fruits with discontinuous ridges and smooth surface	A	IARI, New Delhi
40	G-41-1	Dark green, long fruits with discontinuous ridges, spiny surface and prominent tubercles	A	IARI, New Delhi
41	G-12	Green, long fruits with discontinuous ridges, spiny surface and prominent tubercles	A	IARI, New Delhi
42	G-60	Green, small fruits with discontinuous ridges and prominent tubercles	B	IARI, New Delhi
43	NDBT-9	Green, medium long fruits with discontinuous ridges, spiny surface and prominent tubercles	A	Faizabad, India
44	Sel-41	Dark green, long fruits with discontinuous ridges, medium vine and prominent tubercles	A	Bangladesh
45	Sel-29	Green, medium fruits with discontinuous ridges, pointed tubercles, medium vine	A	Philippines
46	DBG-5	Creamy white, long fruits with discontinuous ridges, prominent tubercles	A	IARI, New Delhi
47	G-42	Green, medium long fruits with discontinuous ridges and spiny surface	A	IARI, New Delhi
48	G-70	Green, medium long fruits with discontinuous ridges and spiny surface	A	IARI, New Delhi
49	NEH-2	Dark green, medium long fruits with continuous ridges, medium vine	A	Meghalaya, India
50	IC-44418	Light green, medium long fruits with discontinuous ridges and spiny surface	A	NBPGR, New Delhi
51	Sel-2-1	Dark green, extra-long fruits with continuous ridges and smooth surface	A	IARI, New Delhi
52	G-7	Green, medium long fruits with discontinuous ridges and spiny surface	A	IARI, New Delhi
53	Sel-58	Green, medium long fruits with discontinuous ridges and spiny surface	A	China
54	NL-39	Green, medium long fruits with discontinuous ridges, and prominent tubercles	A	Odisha, India
55	DBG-38	Dark green, medium long fruits with discontinuous ridges and prominent tubercles	A	West Bengal, India
56	G-12-1	Creamy white, medium long fruits with discontinuous ridges and spiny surface	A	IARI, New Delhi
57	DBG-7	Light green, medium long fruits with discontinuous ridges, and prominent tubercles	A	IARI, New Delhi
58	NEH-4	Creamy white, medium long fruits with ridges and prominent tubercles	A	Manipur, India
59	Sel-57	Dark green, medium long fruits with smooth tubercles, and discontinuous ridges	A	China
60	IC-68294	Light green, medium long fruits with discontinuous ridges	A	NBPGR, New Delhi
61	Sel-13	Green, medium long fruits with discontinuous ridges and prominent tubercles	A	Philippines

62	G-23	Dark green, medium long fruits with discontinuous ridges, prominent tubercles	A	IARI, New Delhi
63	G-73	Green, medium long fruits with discontinuous ridges and spiny surface	A	IARI, New Delhi
64	IC-39725	Green, medium long fruits with discontinuous ridges, spiny surface and prominent tubercles	A	NBPGR, New Delhi
65	G-22	Glossy green, medium long fruits with continuous ridges, and smooth surface	A	IARI, New Delhi
66	IC-68314	Green, extra-long fruits with discontinuous ridges, prominent tubercles and spiny surface	A	NBPGR, New Delhi
67	IC-85643	Green, medium long fruits with discontinuous ridges and spiny surface	A	NBPGR, New Delhi
68	IC-315350	Green, long fruits with continuous ridges and smooth surface	A	NBPGR, New Delhi
69	G-35	Dark green, medium long fruits with discontinuous ridges and smooth surface	A	IARI, New Delhi
70	Sel-31-1	Creamy white, medium long fruits with ridges, prominent tubercles and medium vine	A	China
71	IC-44413	Light green, medium long fruits with discontinuous ridges and spiny surface	A	NBPGR, New Delhi
72	G-47	Dark green, medium long fruits with discontinuous ridges and prominent tubercles	A	IARI, New Delhi
73	CBM-12	Green, extra small fruits with discontinuous ridges, prominent tubercles and spiny surface	B	USA
74	Kalyanpur Baramati	Dark green, long fruits with discontinuous ridges, prominent tubercles and smooth surface	A	West Bengal, India
75	Pusa Do Mausumi	Fruits glossy, green with continuous ridges with smooth surface and vine vigorous long	A	IARI, New Delhi
76	G-71	Dark green, long fruits with spiny surface, prominent tubercles	A	IARI, New Delhi
77	DBGS-21-06	Dark green, long fruits with discontinuous ridges, predominantly gynoecious and prominent spines	A	IARI, New Delhi
78	DBGSG-48-00	Green, long fruits with discontinuous ridges, predominantly gynoecious and prominent spines	A	IARI, New Delhi
79	Punjab-14	Green, medium fruits with continuous ridges and smooth surface	A	Punjab, India
80	DBG-52	Dark green, extra-long fruits with discontinuous ridges	A	West Bengal, India
81	Sel-30-2	Light green, medium long fruits with discontinuous ridges	A	Philippines
82	IC-541249	Dark green, medium long fruits with discontinuous ridges and spiny surface	A	NBPGR, New Delhi
83	IC-469518	Dark green, medium long fruits with discontinuous ridges and spiny surface	A	NBPGR, New Delhi
84	G-5	Dark green, long fruits with continuous ridges and smooth surface	A	IARI, New Delhi
85	MC-84-1	Green, medium long fruit with prominent spines	A	Kerala, India
86	IC-47035	Green, medium long fruits with discontinuous ridges and spiny surface	A	NBPGR, New Delhi
87	Sel-53	Light green, long fruits with continuous smooth ridges	A	Taiwan
88	Andhra Collection	Light green, medium long fruits with discontinuous ridges	A	Andhra Pradesh, India
89	DBG-33-1	Green, medium long fruits with discontinuous ridges and prominent tubercles	A	West Bengal, India
90	Nakhra Local	Medium vine, long, dark green fruits with continuous ridges	A	Odisha, India
91	G-11	Dark green, long fruits with discontinuous ridges and prominent tubercles	A	IARI, New Delhi
92	IC-113875	Green, medium long fruits with discontinuous ridges	A	NBPGR, New Delhi
93	G-43	Green, medium long fruits with discontinuous ridges and spiny surface	A	IARI, New Delhi
94	IC-505629	Light green, medium long fruits with discontinuous ridges and prominent spines	A	NBPGR, New Delhi

95	IC-858650	Dark green, medium long fruits with discontinuous ridges and prominent spines	A	NBPGR, New Delhi
96	G-39	Dark green, small fruits with discontinuous ridges and prominent tubercles	B	IARI, New Delhi

A= M. charantia var. charantia; B= M. charantia var. muricata.
NBPGR, New Delhi: National Bureau of Plant Genetic Resources, New Delhi (Collected from different parts of the country by NBPGR, New Delhi).
IARI, New Delhi: Indian Agricultural Research Institute, New Delhi (Developed advance breeding lines).

Table S2. Forward and reverse primers of 33 SSR markers used in the study.

Sr. No.	Markers	Forward primer	Reverse primer	Tm (°C)	Allele size range (bp)
1	AVRDC_BG-83	TATGCAGGGAAGACTGATGG	TTTTGCTGGCTAAGGTGTTG	50.0	60-100
2	AVRDC_BG-66	AGAGGTCTGCCTCTTCCAAA	CAAGGAACGCAGAAATCCTA	50.0	100-140
3	AVRDC_BG-74	AACACCTTCTGACTCCACCC	CGTTCAATCCTCTCCTCCTC	52.0	100-140
4	AVRDC_BG-1	CAAGGAACGCAGAAATCCTA	GAGGTCTGCCTCTTCCAAAA	50.0	100-140
5	AVRDC_BG-2	GAGCACACAGAAAATTGGGT	TGATCCACTCCCAATCTTAGC	51.0	100-140
6	AVRDC_BG-95	GTTCTCAATTGCATCCGCTA	CAGCAACAGCAACAGCAGTA	50.0	190-260
7	BGSSR-08	TTAGCTCGCAGTCGCCAC	ATTGGCCTCAGCAGCCAG	52.0	390-420
8	CMBR-57	GAAGCACAATCACTCGTTGC	GAACGGGTGTTACCTGAGGA	55.0	120-140
9	CMBR-22	CACGGAAACATCCGACCTAT	TTTTGGGGAATATGGGTTGA	52.0	100-140
10	CMBR-30	ACAGAGCGTAGGCTTGCTTT	ATTGGAGGGCAAGTCTGGT	55.0	270-280
11	CMBR-31	TCTTTTTCATTCCCCCTTTG	GAACTGCACGGAGTTGATGA	52.0	220-240
12	JY-003	GTGGGTGCAATGGGTGTC	CTGCTGCTGTTGCTTCTTC	56.0	50-140
13	JY-004	GTCAACTGCCATCGGTAC	AGGGAAGAAGAAGAAGAAG	56.0	50-80
14	JY-005	TTTATAGCAAACGGCTCA	RGAACATATCGCAAACCTTA	56.0	260-270
15	JY-008	CTCGAACTTTCTGCTC	TGAATTGAATTGCTCT	54.0	260-280
16	JY-009	TAAACAACAAAACCAC	CTCAGAGTCAGAGCAA	52.0	250-270
17	JY-011	AAGTTGGGTTTACGAGTG	TGGATGATGTAGGGTTTC	52.0	170-220
18	McSSR-18	TAAAGAATCGGCCAGTTCGG	GGGGTTAGAGAAAATGAGAGGC	58.0	80-90
19	McSSR-11	TCGTTGTTTCTCCCTCTCTCG	GCATAACACAGAATTGAGGGACC	49.0	70-100
20	McSSR-22	CCATGACCGATGTAGCACTCC	TCGAACCAACCTAAACCAG	54.0	120-140
21	McSSR-47	TTGATTTTGAATCAGCGTTGT	ATTTTGCACAAGGCCTACCA	51.0	180-200
22	McSSR-54	CCATCCATATCCCAATTCCA	TCATCACAAACCTCCCTTTTTC	46.0	120-130
23	N-1	GTCTTCCAGGTTGGGAACAG	ATCTGGTTCCTCGGGAGATT	58.0	110-140
24	N-6	GGGAATTCTCAAAGAGCCAGA	TGGCACACTCTGCATGAAAT	58.0	80-130
25	N-9	ATCCATCCCCACAAGTTGAA	CCATAAGGATATGTTTGCATGG	56.0	40-70
26	S-9	TTCCCATTCACAGATCACTCC	CCACCAAATTCAAGAACCCAC	58.0	270-290
27	S-12	GACATCCTTCTTGCCTCTTACA	GAAACGGAACGAAACCTCA	58.0	100-150
28	S-13	TTGGTTGTGGTGCTGAGTTC	GATGTAGGGGTTGGGTTGAT	58.0	220-260
29	S-20	CCCCTTCTAATCACAACCAA	GGCCTAATTTCTGCCCTTT	57.0	270-290
30	S-24	GCTCTGCGTTTCATTCTTCA	TGAACCCTCAGACTCAAACTC	56.0	250-290

31	S-32	CTAAATCACGCAAACCCATC	GAGCAAAAGACTGAGGAAAACT	56.0	130-180
32	S-33	ATTTAGTGGGGCGGGTAGT	TGGATGAGCATGTTAGGGATC	58.0	220-240
33	S-26	GAACGCCCTGTG CTTTAGC	TTTCGTCTTCCAATGAGCC	57.0	140-180

Tm: Annealing temperature.

Table S3. Pearson Correlation Matrix (Genotypic Correlations Matrix) among different quantitative traits in 96 bitter gourd genotypes.

	AFW (g)	DFFFO	DFFH	DFMFO	FD (cm)	FL (cm)	NNFFF	NNFPP	VL (cm)	YPP (g)
**AFW (g)**	1	-0.124^{NS}	-0.122^{NS}	-0.110^{NS}	0.520**	0.754**	-0.127^{NS}	-0.348**	0.441**	0.653**
**DFFFO**		1	0.801**	0.930**	-0.089^{NS}	0.001^{NS}	0.706**	-0.490**	-0.143^{NS}	-0.560**
**DFFH**			1	0.816**	-0.031^{NS}	-0.019^{NS}	0.614**	-0.475**	-0.059^{NS}	-0.569**
**DFMFO**				1	-0.121^{NS}	-0.036^{NS}	0.553**	-0.413**	-0.163^{NS}	-0.454**
**FD (cm)**					1	0.289*	-0.087^{NS}	-0.408**	0.085^{NS}	0.137^{NS}
**FL (cm)**						1	-0.054^{NS}	-0.223*	0.611**	0.591**
**NFFF**							1	-0.371^{NS}	-0.172^{NS}	-0.488**
**NFPP**								1	-0.029^{NS}	0.363**
**VL (cm)**									1	0.466**
**YPP (g)**										1

** Significant at 1% level of probability. NNFFF: Node number of first female flower; DFFFO: Days to first female flower opening; DFMFO: Days to first male flower opening; DFFH: Days to first fruit harvest; FL: Fruit length (cm); FD: Fruit diameter (cm); AFW: Average fruit weight (g); NFPP: Number of fruits per plant (No.); YPP: Yield per plant(g); VL: vine length(cm).

Table S4. Estimates of direct and indirect effects of different quantitative traits on fruit yield per plant in bitter gourd genotypes.

	AFW (g)	DFFFO	DFFH	DFMFO	FD (cm)	FL (cm)	NNFFF	NNFPP	VL (cm)
**AFW (g)**	**0.6604**	-0.0818	-0.0807	-0.0727	0.3431	0.4979	-0.0841	-0.2296	0.2913
**DFFFO**	0.0264	**-0.2129**	-0.216	-0.198	0.0189	-0.0001	-0.1504	0.1042	0.0305
**DFFH**	0.0163	-0.1353	**-0.1333**	-0.1088	0.0041	0.0025	-0.0818	0.0634	0.0079
**DFMFO**	-0.0189	0.1599	0.1403	**0.172**	-0.0209	-0.0062	0.0951	-0.071	-0.0281
**FD (cm)**	-0.0402	0.0069	0.0024	0.0094	**-0.0775**	-0.0224	0.0068	0.0316	-0.0066
**FL (cm)**	0.141	0.0001	-0.0035	-0.0068	0.0541	**0.187**	-0.0101	-0.0417	0.1142
**NFFF**	0.0096	-0.0532	-0.0463	-0.0417	0.0066	0.0041	**-0.0754**	0.0279	0.013
**NFPP**	-0.167	-0.2351	-0.2283	-0.1983	-0.196	-0.1072	-0.1781	**0.4803**	-0.0137
**VL (cm)**	0.0254	-0.0083	-0.0034	-0.0094	0.0049	0.0352	-0.0099	-0.0016	**0.0577**
**Corelation on YPP (g)**	0.653**	-0.5597^{NS}	-0.5688**	-0.4543**	0.1373^{NS}	0.5908**	-0.4879**	0.3635**	0.4662**

Residual effect = 0.337: Diagonal bold figures represent the direct effect; ** Significant at 1% level of probability. NNFFF: Node number of first female flower; DFFFO: Days to first female flower opening; DFMFO: Days to first male flower opening; DFFH: Days to first fruit harvest; FL: Fruit length (cm); FD: Fruit diameter (cm); AFW: Average fruit weight (g); NFPP: Number of fruits per plant (No.); YPP: Yield per plant(g); VL: vine length(cm).

Table S5. Corresponding Eigen vectors for different principal components (PCs) based on analysis of phenotypic traits.

		PC1	PC2	PC3	PC4	PC5	PC6	PC7	PC8	PC9	PC10
**1**	**NNFFF**	0.36259	0.10837	0.06827	0.07563	-0.80898	0.38508	0.17616	0.0779	-0.03326	0.07386
**2**	**DFFFO**	0.44192	0.18951	0.16971	0.15954	0.19351	0.07439	-0.08469	-0.2383	-0.30184	-0.71867
**3**	**DFMFO**	0.40745	0.15999	0.17619	0.29606	0.32214	-0.13896	0.34468	0.62747	0.17887	0.14861
**4**	**DFFH**	0.43164	0.19301	0.13939	-0.02553	0.26498	0.15444	-0.18789	-0.51178	0.04885	0.60223
**5**	**FL (cm)**	-0.16423	0.49827	0.23029	0.08136	-0.13625	-0.05583	-0.73435	0.31755	0.05444	0.01876
**6**	**FD (cm)**	-0.07347	0.30499	-0.67784	0.05649	0.25118	0.58705	-0.00966	0.16164	-0.07032	-0.00681
**7**	**AFW (g)**	-0.19949	0.50777	-0.10321	0.27041	-0.11343	-0.21262	0.29931	-0.37455	0.55514	-0.1499
**8**	**NNFPP**	-0.22857	-0.36157	0.39731	0.38422	0.14409	0.57259	-0.08759	-0.03703	0.38038	-0.09586
**9**	**YPP (g)**	-0.4016	0.21184	0.23335	0.43681	0.01453	0.01593	0.27071	-0.08996	-0.64184	0.23405
**10**	**VL (cm)**	-0.18766	0.33877	0.42497	-0.67761	0.13642	0.28629	0.31271	0.06917	0.02568	-0.08034

Number	Eigenvalue	Percent		Cum Percent	ChiSquare	DF	Prob>ChiSq
PC1	4.0533	40.533	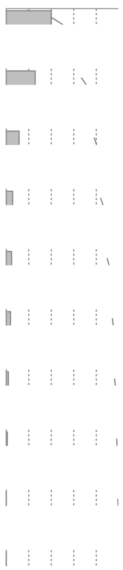	40.533	757.622	44.383	<.0001*
PC2	2.6557	26.557		67.091	539.704	41.414	<.0001*
PC3	1.1673	11.673		78.763	318.569	36.115	<.0001*
PC4	0.5867	5.867		84.630	218.438	28.875	<.0001*
PC5	0.5375	5.375		90.005	185.113	21.795	<.0001*
PC6	0.4460	4.460		94.465	139.575	15.780	<.0001*
PC7	0.2503	2.503		96.968	79.771	10.495	<.0001*
PC8	0.1882	1.882		98.850	48.668	6.084	<.0001*
PC9	0.0705	0.705		99.555	4.547	2.956	0.2029
PC10	0.0445	0.445		100.000	.	0.067	.
